# Increased Chondroprotective Effect of Combining Hyaluronic Acid with a Glucocorticoid Compared to Separate Administration on Cytokine-Treated Osteoarthritic Chondrocytes in a 2D Culture

**DOI:** 10.3390/biomedicines10071733

**Published:** 2022-07-18

**Authors:** Christoph Bauer, Lukas B. Moser, Vivek Jeyakumar, Eugenia Niculescu-Morzsa, Daniela Kern, Stefan Nehrer

**Affiliations:** 1 Center for Regenerative Medicine, Department for Health Sciences, Medicine and Research, University for Continuing Education, 3500 Krems, Austria; lukas.moser@donau-uni.ac.at (L.B.M.); vivek.jeyakumar@donau-uni.ac.at (V.J.); eugenia.niculescu-morzsa@donau-uni.ac.at (E.N.-M.); daniela.kern@donau-uni.ac.at (D.K.); stefan.nehrer@donau-uni.ac.at (S.N.); 2 Department of Orthopedics, University Hospital Krems, Mitterweg 10, 3500 Krems, Austria

**Keywords:** glucocorticoid, hyaluronic acid, inflammation, chondrocyte, osteoarthritis, viscosupplementation

## Abstract

Intra-articular injections of glucocorticoids (GC) or hyaluronic acid (HA) are commonly used interventions for patients suffering from knee osteoarthritis (OA). Both substances are combined to achieve a chondroprotective and anti-inflammatory effect. Clinical studies have shown benefits, but data on the cellular level are still lacking. This study aimed to investigate the effect of the GC triamcinolone hexacetonide, HA, and a mix of both substances on cytokine-treated chondrocytes in vitro. Chondrocytes isolated from human articular cartilage were seeded on 6- and 24-well plates. Mimicking OA’s inflammatory state, cells were treated with IL-1β and IL-17 for six days, whereby, after three days, test substances (10%) were added to the culture medium. Chondrocytes were analyzed on days three and six concerning their actin polymerization, expression of anabolic and catabolic genes, metabolic activity, cytokine release, and reactive oxygen species (ROS). Adding HA or GC/HA to the inflammatory culture medium increased the metabolic activity of chondrocytes, while groups containing GC reduced catabolic gene expression and the release of TNF-α. In addition, enhanced F-actin content was shown supplementing HA or GC/HA to the culture medium. Supplementing GC with HA leads to an anti-inflammatory and chondroprotective effect by diminishing the side effects of GC supplementation alone.

## 1. Introduction

Osteoarthritis (OA) of the knee is a very common disease characterized by inflammation and degeneration of the joint [1]. In addition to cartilage, bone, and synovium, other structures such as the infrapatellar fat pad and the menisci are increasingly recognized as potential biological factors in disease progression and symptomatology [2]. Being a chronic disease, OA patients suffer from ongoing pain, disability, and reduced quality of life, which further deteriorates [3]. The cartilage breakdown is accompanied by a synovial inflammation that plays a significant role in OA progression [4]. Macrophages, lymphocytes, and chondrocytes produce inflammatory cytokines, such as interleukin-1β (IL-1β) or IL-17, and proteolytic mediators such as matrix metalloproteinases (MMPs) [5], leading to an imbalance in metabolic homeostasis and thereby stimulating the degrading process [6,7]. In mild OA, lifestyle alterations, physical therapy, and painkillers are widely used. However, these therapies only treat the symptoms and have no chondroprotective effect [8]. The cartilage is wholly worn in end-stage OA, and the subchondral bone is exposed.

As such patients cannot benefit from chondroprotective therapies, knee arthroplasty remains the treatment of choice. In the best case, patients have increased mobility and decreased pain levels after surgery, leading to improved quality of life [9,10]. With increasing life expectancy and the limited lifetime of the implants, chondroprotective approaches to postponing surgery have been extensively researched. Intra-articular injections of various drugs, preferably used in patients with moderate OA, offer a promising approach. They are administered locally, targeting the chondrocytes directly while limiting systemic side effects [11]. Glucocorticoids reduce inflammation and pain very effectively [12]. However, studies have shown the long-term pro-apoptotic effects of glucocorticoid-treated chondrocytes. Local anesthetics offer a short-term reduction in pain perception but show long-term chondro-destructive potential after multiple injections [13,14]. Hyaluronic acid (HA) has become very popular in recent years as it operates in multiple ways. HA can reduce the inflammatory process, albeit lesser than glucocorticoids. By stimulating the synthesis of endogenous HA, intra-articular injected HA increases lubrication and improves viscoelastic properties [15].

In osteoarthritic chondrocytes, HA increases metabolic activity and thereby biosynthesis on a cellular level [16,17]. All these various effects lead to a reduction in pain and an increase in function of the patients [15]. Recently, efforts have been made to combine glucocorticoids and HA to achieve synergistic chondroprotective effects. Glucocorticoids could strengthen the weaker anti-inflammatory effects of HA, and the chondroprotective effect of HA could mitigate the pro-apoptotic chondro-destructive effects of glucocorticoids. First clinical studies have confirmed the benefits of combining HA and glucocorticoids, showing decreased pain levels in patients suffering from knee OA compared to separate administration of either glucocorticoids or HA [18,19,20,21]. However, although these clinical data are very promising, no experimental studies confirm the beneficial effects on a cellular level.

The current study compares the chondroprotective effects of combining HA and glucocorticoids with separate administration of HA or glucocorticoids on cytokine-treated human chondrocytes in a 2D culture.

We hypothesized that the HA in the mixed formulation confers a chondroprotective benefit compared with the glucocorticoid and the HA injection alone.

## 2. Materials and Methods

### 2.1. Isolation and Cultivation of Human Chondrocytes

Human articular cartilage was received from five osteoarthritic patients undergoing total knee arthroplasty at a local University Hospital. All patients suffered from end-stage knee OA for one to six years. Radiological degenerative signs combined with the clinical symptoms set up the indication for total knee arthroplasty. Patients who received systemic anti-inflammatory drugs within six months before surgery or/and patients that had operative cartilage treatment were excluded from this study. Informed consent was obtained in all cases, and this study was approved by the regional ethical committee (GS1-EK-4/665-2020). During surgery, the cartilage/bone pieces were stored in a sterile cup containing phosphate-buffered saline (PBS) supplemented with antibiotics (penicillin 200 U/mL; streptomycin 0.2 mg/mL) and Amphotericin B (2.5 μg/mL; Sigma-Aldrich Chemie GmbH, Steinheim, Germany) before transportation to the research facility. For chondrocyte isolation, articular cartilage was minced into 2 mm^3^ pieces prior to enzymatic digestion with Liberase TM (0.2 WU/mL, Roche Diagnostics GmbH, Mannheim, Germany) in medium (GIBCO^®^ DMEM/F12 GlutaMAX™-I, Invitrogen, LifeTech Austria, Vienna, Austria) supplemented with antibiotics and Amphotericin B. After 18–22 h at 37° under permanent agitation, undigested debris was removed by passing the chondrocyte suspension through a Cell Strainer with 40 μm pores (BD, Franklin Lakes, NJ, USA). Subsequently, cells were washed with medium, centrifuged (10 min, 500× *g*, room temperature [RT]), and resuspended in a growth medium supplemented with antibiotics, Amphotericin B, 10% Fetal Calf Serum (FCS; GIBCO^®^ by Life Technologies, Carlsbad, CA, USA) and 0.05 mg/mL ascorbic acid (Sigma-Aldrich Chemie GmbH, Steinheim, Germany)). Cell viability was determined with trypan blue staining (Sigma-Aldrich Chemie GmbH, Steinheim, Germany). Cells were counted using a hemocytometer.

For expansion, the isolated cells (P0) were seeded in growth medium in 75 cm^2^ culture flasks (Nunc, Rochester, NY, USA) with a density of 1 × 10^4^ cells/cm^2^. Cells were cultivated at 37° in a humified environment with 5% CO_2_. The medium was changed every two to three days until 80% confluency was achieved. Following expansion, the chondrocytes were harvested using accutase (1.5 mL/flask; Sigma-Aldrich Chemie GmbH, Steinheim, Germany) and counted as described above. In addition, cells (P1) were seeded (1.4 × 10^4^ cells per cm^2^) in 6-well plates for microscopy, gene expression, and quantification of sulfated glycosaminoglycans (sGAG) and in 24-well plates for measuring the metabolic and ROS activity of the chondrocytes as well as determination of cytokines in the supernatant. In addition, phalloidin staining of chondrocytes was performed in chamber slides (8-well; 1 × 10^3^ cells per well) to investigate filamentous actin (F-actin).

### 2.2. Treatment of Chondrocytes

After seeding, cells were pre-incubated for three days to ensure cell attachment before treatment with IL-1β (1 ng/mL; Sigma-Aldrich, St. Louis, MO, USA) and IL-17 (2 ng/mL; Sigma-Aldrich, St. Louis, MO, USA) was performed. The treatment lasted for six days, with medium change (supplemented with cytokines) and addition of the test substances glucocorticoid (triamcinolone hexacetonide, Riemser Pharma GmbH, Austria), hyaluronic acid (Anika Therapeutics Inc., Bedford, MA, USA), and glucocorticoid + hyaluronic acid (Anika Therapeutics Inc., Bedford, MA, USA) on day 3. In total, five groups were observed, as shown in Table 1:

On days 3 and 6, chondrocytes were analyzed for metabolic activity and ROS production, and RNA isolation for gene expression was performed. Supernatants were stored at −80 °C for further analysis of sGAG and cytokines. After six days, chondrocytes cultivated in chamber slides were fixed and stained for DAPI (nucleus) and phalloidin (F-actin).

### 2.3. Metabolic Activity

Metabolic activity of the chondrocytes was measured after three and six days using an XTT-based ex vivo toxicology assay kit according to the manufacturer’s instructions (Cell Proliferation Kit II, Roche Diagnostics, Basel, Switzerland). Briefly, XTT solution (245 µL of XTT reagent and 5 µL of activation reagent) was added to each of the 24-wells with a 500 µL culture medium, followed by a 4 h incubation period at 37 °C in the atmosphere of 95% air and 5% CO_2_. After incubation, absorbance was measured at 492 nm and 690 nm (background wavelength) in duplicates in the 24-well plate using a multimode microplate reader (BioSynergyTM 2, BioTek Instruments, Inc., Winooski, VT, USA) with Gen 5 software. A culture medium with 10% of the various test substances was used as a blank control.

### 2.4. Reactive Oxygen Species (ROS) Activity

To measure ROS activity, a 5 mM DCF-DAH_2_ solution was prepared by dissolving dichlorodihydrofluorescein (H_2_DCF-DA, Invitrogen, Carlsbad, CA, USA) in DMSO (Sigma-Aldrich, St. Louis, MO, USA). First, the culture medium of the 24-well plate was discarded, and cells were washed with 500 µL Dulbecco’s phosphate-buffered saline (DPBS). A volume of 500 µL H_2_DCF-DA solution was added to each well and incubated for 30 min. In the meantime, a 100 µM hydrogen peroxide (H_2_O_2_) solution was prepared as a positive control. After 30 min, H_2_O_2_ was added to the appropriate well, and the whole plate was incubated for additional 15 min. Post-incubation, fluorescence (excitation 485 nm/emission 535 nm) was measured using a multimode microplate reader (SynergyTM 2, BioTek Instruments, Inc., Winooski, VT, USA) with Gen 5 software.

### 2.5. Gene Expression

#### 2.5.1. Total RNA Isolation and Reverse Transcription

RNA isolation of osteoarthritic chondrocytes was performed using the High Pure RNA Isolation Kit (Roche Diagnostics, Basel, Switzerland) following the manufacturer’s protocol. The first two steps were adjusted as RNA from the cells was isolated from the 6-well plate and not from a cell pellet. For this, 400 µL lysis buffer was added to each well of the 6-well plate and shaken for 30 s. Next, the lysed cell suspension was transferred to a spin column (High Pure RNA), while wells were washed with 200 µL PBS and the volume was also pipetted to the spin column. Finally, the remaining steps were performed as in the manufacturer’s protocol.

Complementary DNA (cDNA) from messenger RNA (mRNA) was synthesized using the Transcriptor First Strand cDNA Synthesis Kit (Roche Diagnostics, Basel, Switzerland). RNA from bacteriophage MS2 was added to stabilize isolated RNA during cDNA synthesis.

#### 2.5.2. Quantitative Polymerase Chain Reaction (qPCR)

Probe-based qPCR was performed in triplicates in the LightCycler^®^ 96 using FastStart Essential DNA Probe Master (both from Roche Diagnostics, Basel, Switzerland). Probe/primer pairs (Table 2) were designed for cartilage-specific genes (*COL2A1*, *ACAN*, *PRG4*, *SOX9*) and catabolic genes (*MMP3*, *MMP13*, *NOS2*) with IDT Real-Time qPCR software and synthesized by IDT (Integrated DNA Technologies, Leuven, Belgium). Glyceraldehyde-3-phosphate dehydrogenase (*GAPDH*) mRNA expression level was used as housekeeping gene expression control. The annealing temperature was experimentally determined for reference, and each target gene and the relative expression was evaluated using the R = 2^−ΔCt [Mean target − Mean reference]^ method [22].

### 2.6. Cytokine Quantification

Stored supernatants from cytokine-treated osteoarthritic chondrocytes incubated with various test substances were analyzed for the level of tumor necrosis factor-α [TNF-α] using the Bio-Plex Pro Assay and the Bio-Plex 200 analyzer (Bio-Rad Laboratories, Inc., Hercules, CA, USA). In this cytokine multiplex assay, antibodies are covalently coupled to magnetic beads with a unique fluorescence dye. Thus, the concentrations of each analyte can be determined. The value below the lower limit of detection for the analyte was recorded as the lower limit of quantification (LLOQ). For example, analyzed TNF-α had an LLOQ of 3.33 pg/mL. In addition, the volume of every sample supernatant was measured to quantify proteins.

### 2.7. Sulfated Glycosaminoglycans (sGAG)

The quantification of sGAG was conducted according to Barbosa et al. [23]. In brief, cell culture supernatants were collected after six days and digested overnight using 25 U/mL proteinase K (Sigma, St. Louis, MO, USA) at 56 °C. After enzyme inactivation (90 °C, 10 min), supernatants were transferred to an ultra-free filter reaction tube of 0.1 µm pore size (Millipore, Billerica, MA, USA) and centrifuged (12,000× *g*, 4 min, RT). Next, 100 µL filtrate was vigorously mixed (30 min) with 1 mL of a 1.9-dimethyl-methylene blue solution (DMMB, pH = 3.2) to allow the formation of DMMB and sGAG complexes in the sample. The complexes were pelleted via centrifugation (12,000× *g*, 10 min, RT) and dissolved in a decomplexation 4 M GuHCl solution at pH 6.8 containing 10% propan-1-ol. After 30 min of shaking, the absorbance at 656 nm was measured photometrically using an Ultrospec 3300 pro photometer (Amersham Bioscience plc, Amersham, UK). The sGAG amount was calculated using a standard curve with shark chondroitin sulfate (Sigma, St. Louis, MO, USA). The measurement was performed in duplicates for all conditions of every patient (*n* = 5).

### 2.8. Phalloidin Staining

The culture medium of chondrocytes was discarded, and cells were washed twice with DPBS before a fixation step with 4% formaldehyde for 25 min at RT was performed. After additional washing steps, a 50 mM ammonium chloride solution was used to neutralize the acidity of formaldehyde. Next, incubation was performed for 10 min at RT. Permeabilization of the cells was then achieved using DPBS + 0.1% Triton X-100 for 30 min at RT. Two washing steps were followed by staining with 1 U/mL phalloidin AF488 (stock concentration 200 U/mL; Invitrogen, Carlsbad, CA, USA) for 30 min at 37 °C in the dark. Cells were then washed twice with DPBS + 0.1% Tween-20 and once with DPBS before a 1 µg/mL DAPI solution was added, followed by incubation for 5 min at RT in the dark. Additional washing steps with DPBS + 0.1% Tween-20 (twice) and DPBS (once) followed. After drying the samples at RT in the dark, the mounting medium ProLong^TM^ Gold antifade reagent (1 drop per well; Invitrogen, LifeTech Austria, Vienna, Austria) was added to each well and covered with a cover glass. The mounting medium was left to cure for 24 h in the dark at RT before slides were stored at 4 °C until confocal microscope (Leica TCS SP8 MP, Leica Microsystems, Wetzlar, Germany) investigations.

### 2.9. Statistical Analysis

All statistical analysis was performed using GraphPad Prism Software (Version 9.0, GraphPad Prism Software Inc., San Diego, CA, USA). Data are expressed as the mean ± standard deviation. The statistical analysis was carried out using a one-way analysis of variance (ANOVA). Multiple comparisons were performed, followed by Dunn’s test to correct multiple comparisons. Statistical significance was set at *p* < 0.05 and indicated in the figures as * *p* < 0.05, ** *p* < 0.01, and *** *p* < 0.001.

## 3. Results

### 3.1. Metabolic Activity

**Figure 1:**
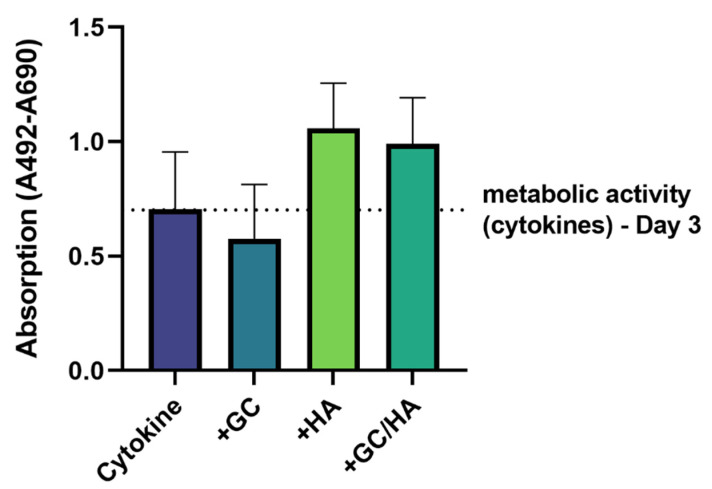
Metabolic activity (XTT measurement) of chondrocytes treated for three days with pro-inflammatory cytokines and another three days with cytokines with and without supplementing the test substance (10%). The dotted line represents the metabolic activity of cytokine-treated chondrocytes after 3 days, before test substances were added.

Chondrocytes displayed elevated metabolic activity levels on day 6 after adding hyaluronic acid-containing test substances after three days of cytokine treatment (Figure 1). In comparison, glucocorticoid supplementation alone resulted in a metabolic activity level similar to cytokine treatment with a mean value lower than on day 3.

### 3.2. ROS Activity

**Figure 2:**
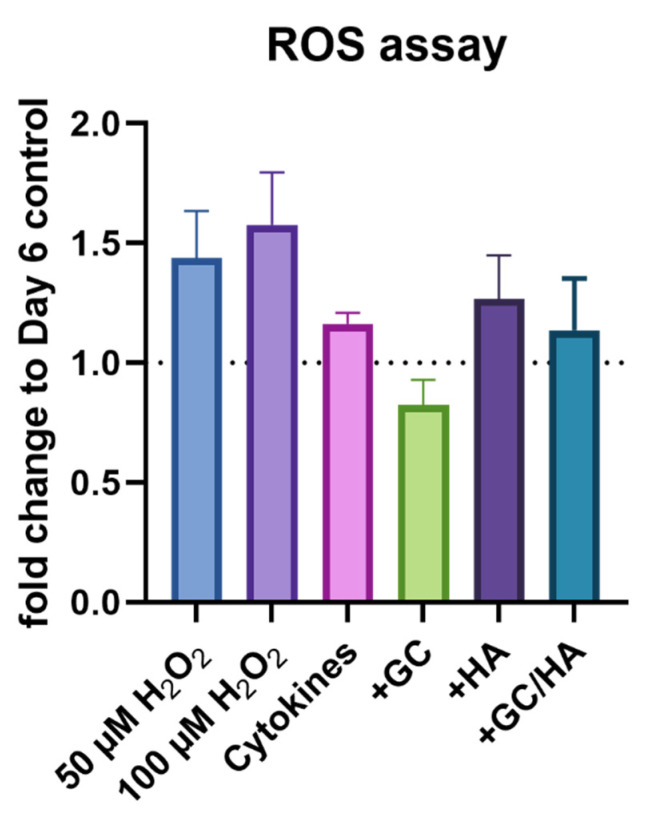
Measurement of ROS after a 3-day incubation with cytokines followed by 3-day treatment with cytokines with and without supplementing test substances (10%). In addition, two positive controls were analyzed with H_2_O_2_. The dotted line represents the control group on day 6.

Measurement of reactive oxygen radicals was referred to the control group on day 6 (culture medium without cytokines and test substance) as the positive controls (two different H_2_O_2_ concentrations) did not contain cytokines and test substances (Figure 2). The addition of H_2_O_2_ increased ROS activity up to 1.5-fold of the control group, while cytokine treatment alone and the combination of HA or GC/HA showed slightly elevated levels compared to the control group. However, only GC treatment resulted in a lower level of ROS synthesis.

### 3.3. Gene Expression

**Figure 3:**
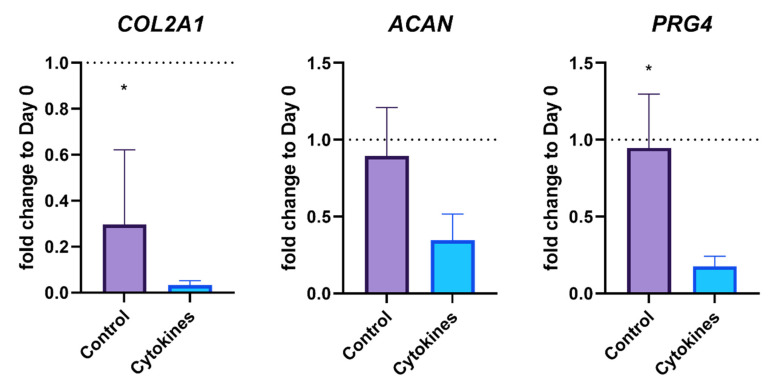
Expression of cartilage-specific genes after three days with and without Il-1ß/IL-17 treatment. The single asterisk indicates *p* < 0.05. The dotted lines represent the initial gene expression on day 0.

**Figure 4:**
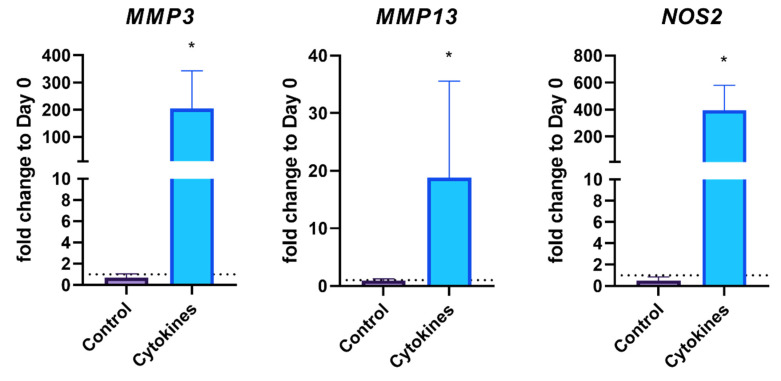
Expression of the catabolic genes *MMP3*, *MMP13* and *NOS2* after three days with and without Il-1ß/IL-17 treatment. The single asterisk indicates *p* < 0.05. The dotted lines represent the initial gene expression on day 0.

The qPCR results on day 3 confirmed that cytokine treatment induces an inflammatory state. As shown in Figure 3, three days after cytokine treatment, the cartilage-specific genes *COL2A1*, *ACAN*, and *PRG4* are significantly reduced compared to the control group. In contrast, genes such as those of *MMP3*, *MMP13* and *NOS2*, indicative of inflammation, were significantly increased (Figure 4). Thus, an inflammatory state was confirmed in the cell culture model.

**Figure 5:**
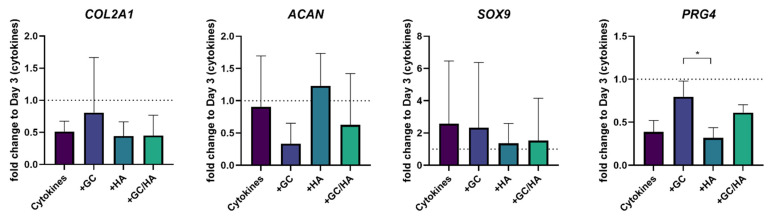
Expression of cartilage-specific genes; shown as fold change to day 3, when added test substances. The single asterisk indicates *p* < 0.05. The dotted lines represent the gene expression of cytokine-treated chondrocytes after 3 days, before test substances were added.

Subsequently, the gene expression value on day 3 was used for the change in the following three days, in which the cells were further cultured with cytokines alone or in combination with 10% glucocorticoid (GC), 10% hyaluronic acid (HA), or 10% glucocorticoid/hyaluronic acid (GC/HA), respectively (Figure 5). Supplementary treatment with cytokines with and without test substances further reduced the expression of *COL2A1*, but there was no significant difference between the groups. The transcription factor of *COL2A1*, *SOX9*, showed high standard deviations with increased expression levels compared to day 3, but without a significant difference between the groups. In contrast, ongoing cytokine treatment did not affect aggrecan expression, as the fold change to day 3 was around 1. Supplementing 10% of HA also kept aggrecan expression at this level. Adding 10% GC or GC/HA reduced aggrecan expression compared to day 3 (cytokine treatment) but without significance. In contrast, *PRG4* showed a reversed expression pattern. Here, GC and GC/HA supplementation led to decreased gene expression but was less intense than in the cytokine and HA group.

**Figure 6:**
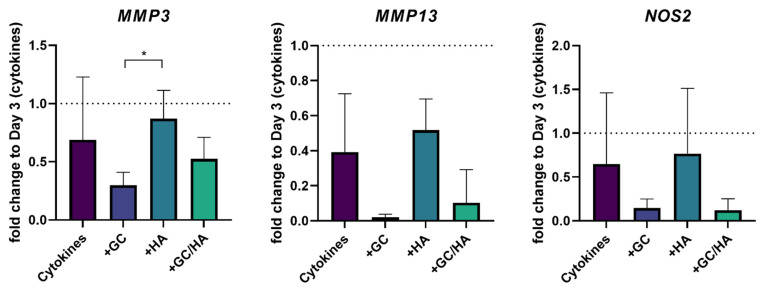
Expression of the catabolic genes *MMP3*, *MMP13* and *NOS2*; shown as fold change to day 3, when test substances (10%) were added. The single asterisk indicates *p* < 0.05. The dotted lines represent the gene expression of cytokine-treated chondrocytes after 3 days, before test substances were added.

Figure 6 shows that expression levels of the catabolic genes *MMP3* and *MMP13*, as well as *NOS2*, responsible for nitric oxide production from L-arginine, could be significantly reduced using GC or GC/HA. However, cytokine treatment alone and the HA group had much higher expression levels of these genes.

### 3.4. Cytokine Release

**Figure 7:**
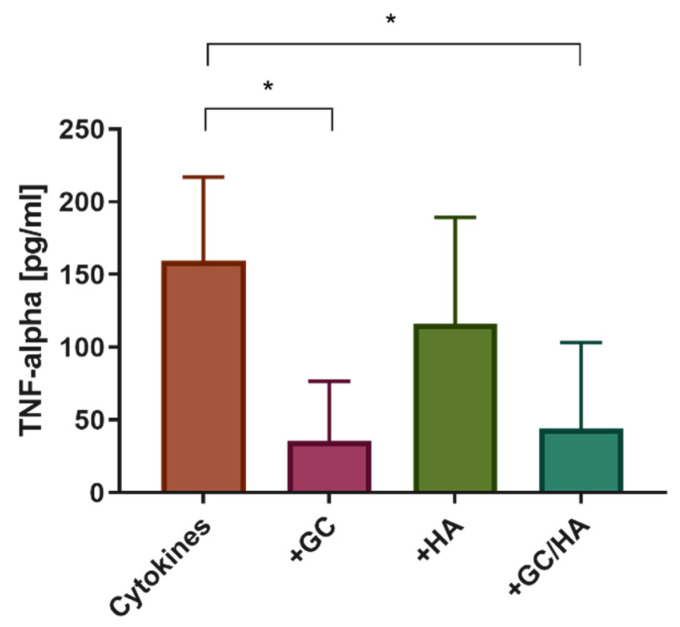
Measurement of TNF-α in the supernatant of treated chondrocytes. The single asterisk indicates *p* < 0.05.

Treatment of human osteoarthritic chondrocytes with IL-1β and IL-17 starting on day 3 showed significantly increased release of the pro-inflammatory cytokine TNF-α on day 6 in the positive control group (cytokines without test substance) compared with the GC and GC/HA group (Figure 7). The addition of 10% HA also showed an increased level of TNF-α release, but without significance to the two groups with GC in the test substance. In the control group (not shown) no TNF-α release was detectable (<OOR).

### 3.5. Sulfated Glycosaminoglycans (sGAG)

**Figure 8:**
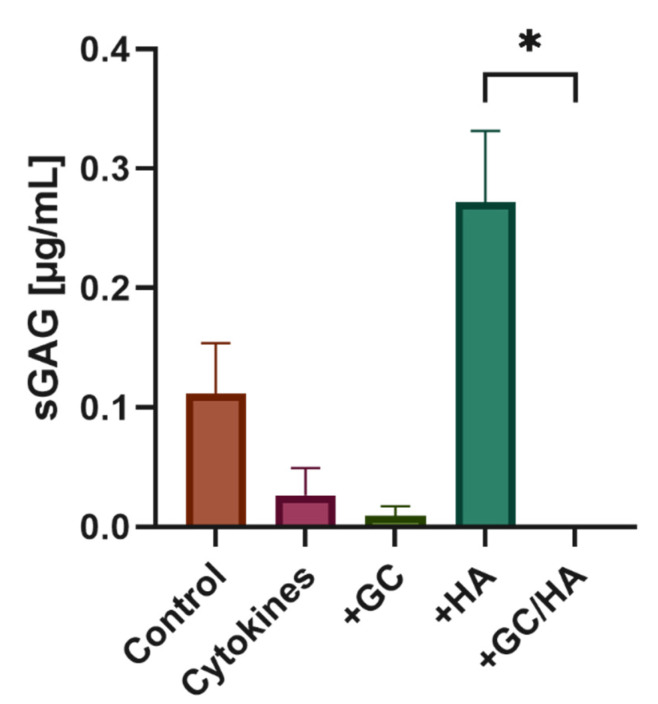
Concentration of sulfated glycosaminoglycans in the culture medium on Day 6 after adding 10% of test substances to cytokine-treated chondrocytes. The single asterisk indicates *p* < 0.05.

Figure 8 shows that treating human osteoarthritic chondrocytes with cytokines leads to a decrease in the synthesis of sulfated glycosaminoglycans after six days compared with the control group. Adding 10% GC or 10% of the GC/HA combination to cytokine-treated chondrocytes decreased sGAG concentration with a not measurable amount in the GC/HA group. In contrast, when only 10% HA is added to the cytokine medium, the sGAG concentration increases significantly compared with the other substances and cytokine treatment alone. The sGAG concentration was also increased by approximately 2.5 fold compared to the control group.

### 3.6. F-Actin Staining

**Figure 9:**
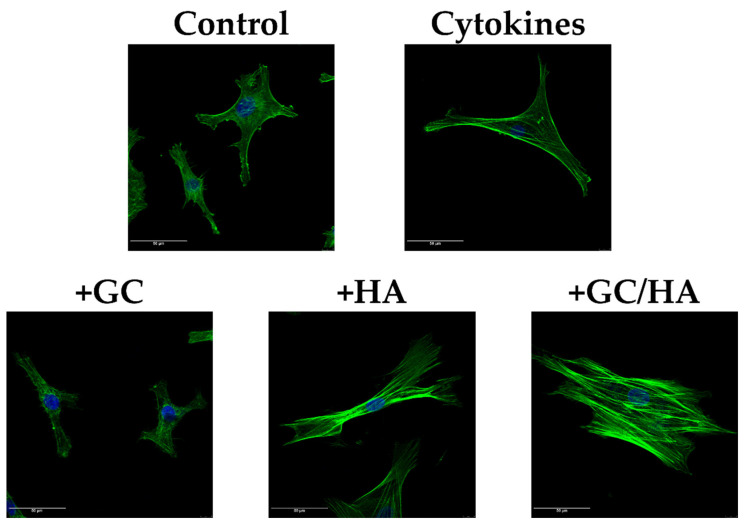
Confocal microscope images showing F-actin staining (green, phalloidin) and the nucleus (blue, DAPI). Scale bar 50 µm.

On day 6, chondrocytes of the control group and cells subjected to treatment with pro-inflammatory cytokines and 10% of the test substances were stained for their F-actin content and analyzed by confocal microscopy as shown in Figure 9. Significant differences between the individual conditions were shown. Most cells showed a typical spread morphology with thick fibrils in the control group. F-actin staining was used as the basis for the other conditions. Chondrocytes treated with cytokines were spread out as in the control group but with slightly thinner fibrils and cortically localized stress fibers. In addition, the cells appeared starved with a lower level of F-actin. This low F-actin content was also seen in chondrocytes treated with 10% GC. Here, a slight collapse of the actin structure can also be seen. The 10% addition of test substances with HA to the cytokine-treated chondrocytes tendentially increased the cell volume on the one hand, but also the F-actin content with additional forming of stress fibers. This was also shown in the GC/HA group, where more stress fibers were formed.

## 4. Discussion

Our current study investigates the effects of a glucocorticoid (GC, triamcinolone hexacetonide), hyaluronic acid (HA), and a combination of both products on osteoarthritic (OA) chondrocytes post-treatment with IL-1β and IL-17 for three days. The in vitro experiments were conducted in a 2D monolayer to examine the adverse effects of GC either being treated alone or in combination with HA. Results indicated that the combination product GC/HA acts similar to GC as an anti-inflammatory substance whilst also diminishing the adverse side effects of GC, corresponding to reduced metabolic activity and altered cytoskeleton appearance.

In our cell culture model, the two pro-inflammatory cytokines, IL-1β and IL-17, were used to trigger inflammation, mimicking osteoarthritis. The pro-inflammatory cytokine IL-1β is one of the central pro-inflammatory cytokines in many diseases and activates various pathways leading to the progression of osteoarthritis [24,25]. IL-17, on the other hand, is crucially involved in changes to the transcriptome of chondrocytes, which has also been shown in studies of osteoarthritis patients [26,27]. In addition, IL-17 leads to an increase in TNF-α production [28], which is shown in our study within the group of simple cytokine treatments. All of these pro-inflammatory cytokines lead to a decrease in cartilage-specific gene expression and an increase in the expression of genes encoding for degradative enzymes [29]. In our study, these gene expression patterns were used to confirm the inflammatory state of the chondrocytes. The metabolic activity of chondrocytes increased when HA was combined with GC, with no difference to treatment with HA alone, compared to treatment with either cytokines or GC. However, the increased metabolic activity of the HA and GC/HA groups showed different patterns in sGAG synthesis. The sGAG synthesis rate by adding HA could not be achieved with GC supplementation. In contrast, there was a significant reduction compared with the HA group, although adding GC alone also vastly decreased sGAG synthesis. The results of HA and GC were consistent with a study by Schaefer et al., which used comparable concentrations of the test substances. In addition, the combination product showed increased sGAG synthesis [30], which could not be shown in our study. The results indicate that high-molecular-weight HA at the used concentration is constructive to alleviate the cytokine-induced proteoglycan catabolism and matrix turnover, as previously reported in a study [31].

Similarly, Siengdee et al. investigated the effect of the glucocorticoid administration of dexamethasone and prednisolone on porcine cartilage explants ex vivo. They identified that the cellular toxicity was higher in the prednisolone treatment group than in treatment with dexamethasone. However, when both glucocorticoids were combined with HA, they exhibited a chondroprotective effect. Furthermore, the release of sGAGs into the culture media was reduced within the dexamethasone/HA group compared with the prednisolone/HA group. A similar trend was observed in the collagen content, indicating that the addition of HA is vital for the chondroprotective effect [32].

Adding test substances to the cytokine-treated chondrocytes should reverse the negative effect on gene expression patterns, as high-molecular-weight HA and GC have anti-inflammatory effects [33]. The former has already been shown in a co-culture study with HA from another manufacturer [31]. In the present study, there was a non-significant difference in the cartilage-specific genes *COL2A1*, *ACAN* and *SOX9*. However, the expression of *ACAN* inclined to reduce further in the test compounds with GC compared with day 3. Song et al. observed a similar decrease after 72 h incubation of chondrocytes with the glucocorticoid dexamethasone [34]. *PRG4*, a component of the extracellular matrix and synovial fluid [35], was maintained at a significantly higher level of gene expression with GC than HA alone, while cytokine treatment also resulted in a decrease.

Treatment with IL-1β and IL-17 also increases the expression of genes for matrix metalloproteinase (*MMP3*, *MMP13*) and inducible nitric oxide synthase 2 (*NOS2*). In combination with HA, the addition of GC showed that these genes were more strongly repressed than without the addition of a test substance or HA supplementation alone. This effect of GC (e.g., dexamethasone or triamcinolone hexacetonide) on catabolic genes has been reported in several studies [36,37]. In comparison, the high-molecular-weight HA used did not show this anti-inflammatory effect, possible due to the wide molecular-weight range Typically, high-molecular-weight HA reduces catabolic gene expression [38]. However, supplementation of HA increased sGAG synthesis and metabolic activity, as mentioned before. In addition, a slight, although not significant, decrease in TNF-α was also shown by the addition of HA. However, this was not reflected in the catabolic gene expression, as TNF-α, as with a variety of other cytokines, leads to increased expression of *MMP3*, *MMP13*, and *NOS2* by chondrocytes [39]. In comparison, supplementation with GC or GC/HA significantly decreased TNF-α levels and gene expression of *MMPs* and *NOS2*. This is supported by other studies [36,37], as the binding of glucocorticoids via GC receptor inhibits the transcription factor NF-κB, which is involved in many biological processes regulating the inflammatory response and cell survival functions [40].

This, in turn, may also impact cytoskeletal components such as F-actin as high levels of inducible nitric oxide synthase lead to the synthesis of nitric oxide (NO), which has been shown to inhibit actin polymerization [41]. Furthermore, the influence of pro-inflammatory cytokines such as IL-1β or TNF-α, as a consequence of inducible nitric oxide synthase, activates different pathways and leads to F-actin reorganization [41,42]. These factors also influence the mechanical properties of the cells, e.g., by forming a large number of so-called stress fibers [43], which may also negatively affect chondrogenic markers (e.g., *COL2A1*, *ACAN*). In our study, cytokine treatment showed an apparent reorganization of the cytoskeleton with cortical stress fibers compared to the control group and a cellular contraction as described by Haudenschild et al. [44]. In contrast, no stress fibers occurred by GC treatment. Therefore, when comparing this group with the control group, it is considered that disruption in the F-actin structure occurred, which could be demonstrated by pharmacological agents in the mesenchymal stem cells. Furthermore, the cell stiffness was reduced due to missing stress fibers [45].

In contrast to GC, the combination with hyaluronic acid showed an opposite effect with increased stress fiber content. This also occurred in the HA group as a possible consequence of chondrocytes binding to their extracellular environment via focal adhesion complexes [46].

Due to pro-inflammatory cytokines, the increased *NOS2* expression subsequently leads to increased NO production, resulting in oxygen radicals (ROS) [47]. Our study showed a trivial fold-change increase in ROS in the cytokine treatment group. This slight increase was also shown by adding the substances HA and GC/HA. Using a glucocorticoid (triamcinolone hexacetonide) alone reduced ROS production in the chondrocytes. This is also consistent with a study by Amin et al., which showed inhibition of *NOS2* expression using glucocorticoids, representing one possibility to reduce ROS production, among many others [48].

Since articular chondrocytes constantly produce ROS in small amounts to maintain cartilage homeostasis, osteoarthritic chondrocytes levels are much higher and could have already reached a certain plateau in the exposure to pro-inflammatory factors [49]. Indeed, a correlation between OA and increased oxidative stress or ROS could be shown [50,51].

Optimizing conservative treatment of patients suffering from early to moderate knee osteoarthritis is incredibly clinically relevant. Local anesthetics (LA) and GC are most frequently administered together due to their favorable painkilling and anti-inflammatory properties. However, intra-articular injection of both substances (LA and GC) affects the breakdown of the collagen fragments resulting in degradation and joint space narrowing of the cartilage over time, leading to loss of polysaccharides and a reduction in surface lubrication [52,53]. On the other hand, HA administered alone acts as a viscosupplement, increases lubrication, and reduces friction. Therefore, combining GC with HA offers synergistic potential as it maintains the positive effects of both substances and mitigates the adverse effects of GC. Furthermore, clinical studies and meta-analyses have shown that GC/HA reduces pain and improves clinical function more effectively than HA injections alone [54,55].

In our study, a few limitations can be noted. First, this study was carried out using a 2D culture of osteoarthritic chondrocytes. This cultivation method reduces the chondrocyte phenotype progressively [56]. For this reason, only passage 1 (P1) cells were used in this study. Second, the cytokine concentration used for the treatment of chondrocytes is very high and not comparable to native conditions, where the degradative processes occur over months and years. In addition, only two cytokines were used in this study. In osteoarthritis, many other cytokines (e.g., IL-6, TNF-α, IL-15) also play an important role during the progression of the disease [6]. In addition, no anti-inflammatory cytokines were analyzed. This could have given us further information on whether the implemented hyaluronic acid has anti-inflammatory properties or not. Another aspect is the cell culture model, as cells are not directly exposed to the substances under normal conditions. Here, further studies should be performed using different cultivation methods (e.g., explant cultures and 3D cultures).

## 5. Conclusions

Cytokine treatment of osteoarthritic chondrocytes with 10% GC or a combination of GC and HA showed an anti-inflammatory effect, as TNF-α release was significantly reduced and gene expression patterns of catabolic enzymes tended to be decreased. In contrast, supplementation of HA alone did not show an anti-inflammatory effect, but when combined with GC, it reduced the adverse effects of GC (e.g., decreased metabolic activity or low F-actin content with collapsed structures). In addition, a significantly increased sGAG concentration by supplementation with 10% HA compared with the combination product was shown, and HA in the test substance tended to increase metabolic activity. However, the present cell culture model could not detect an apparent effect on anabolic gene expression and ROS activity. Thus, our future aims are to adapt the culture model and expand experiments to an explant culture model, generating more native conditions. In conclusion, combining HA with the GC triamcinolone hexacetonide can partially combine the beneficial properties of both compounds and minimize their negative influences on cells.

## Figures and Tables

**Table 1:** Test groups and their cultivation conditions with or without cytokines and test substances.

Group	Cytokines (IL-1β, IL-17)	Test Substance
Control	−	−
Cytokines	+	−
GC	+	+10% glucocorticoid (4.5 mg/mL)
HA	+	+10% hyaluronic acid (22 mg/mL, MW between 1 and 2.9 × 10^6^ Da, ultra-pure and non-avian)
GC/HA	+	+10% glucocorticoid/hyaluronic acid

**Table 2:** Sequences of Primers used in qPCR.

Gene	Identification	Sequence (3′–5′)	
		Sense	Antisense
Glyceraldehyde-3-phosphate dehydrogenase (*GAPDH*)	NM_002046	ACATCGCTCAGACACCATG	TGTAGTTGAGGTCAATGAAGGG
Collagen type II, alpha I (*COL2A1*)	NM_001844	AAGACGTGAAAGACTGCCTC	TTCTCCTTTCTGTCCCTTTGG
Aggrecan core protein I (*ACAN*)	NM_001135	TGTGGGACTGAAGTTCTTGG	AGCGAGTTGTCATGGTCTG
Transcription factor SOX-9 (*SOX9*)	NM_000346	ACTTGCACAACGCCGAG	CTGGTACTTGTAATCCGGGTG
Proteoglycan 4/Lubricin (*PRG4*)	NM_005807	AGAACTGGCCTGAATCTGTG	ACCTGTGTCGTTTCTCCATAC
Matrix metalloproteinase-3 (*MMP3*)	NM_002422	CCAGGGATTAATGGAGATGCC	AGTGTTGGCTGAGTGAAAGAG
Matrix metalloproteinase-13 (*MMP13*)	NM_002427	GATGACGATGTACAAGGGATCC	ACTGGTAATGGCATCAAGGG
Nitric oxide synthase 2 (*NOS2*)	NM_000625	GTTTGACCAGAGGACCCAG	ATCTCCTTTGTTACCGCTTCC

## Data Availability

Not applicable.

## References

[1] Goldring M.B., Otero M. (2011). Inflammation in Osteoarthritis. Curr. Opin. Rheumatol..

[2] Melrose J., Fuller E.S., Little C.B. (2017). The Biology of Meniscal Pathology in Osteoarthritis and Its Contribution to Joint Disease: Beyond Simple Mechanics. Connect. Tissue Res..

[3] Neogi T. (2013). The Epidemiology and Impact of Pain in Osteoarthritis. Osteoarthr. Cartil..

[4] Mathiessen A., Conaghan P.G. (2017). Synovitis in Osteoarthritis: Current Understanding with Therapeutic Implications. Arthritis Res. Ther..

[5] Molnar V., Matišić V., Kodvanj I., Bjelica R., Jeleč Ž., Hudetz D., Rod E., Čukelj F., Vrdoljak T., Vidović D. (2021). Cytokines and Chemokines Involved in Osteoarthritis Pathogenesis. Int. J. Mol. Sci..

[6] Wojdasiewicz P., Poniatowski Ł.A., Szukiewicz D. (2014). The Role of Inflammatory and Anti-Inflammatory Cytokines in the Pathogenesis of Osteoarthritis. Mediat. Inflamm..

[7] Sokolove J., Lepus C.M. (2013). Role of Inflammation in the Pathogenesis of Osteoarthritis: Latest Findings and Interpretations. Ther. Adv. Musculoskelet. Dis..

[8] Vaishya R., Pariyo G.B., Agarwal A.K., Vijay V. (2016). Non-Operative Management of Osteoarthritis of the Knee Joint. J. Clin. Orthop. Trauma.

[9] Cross W.W., Saleh K.J., Wilt T.J., Kane R.L. (2006). Agreement about Indications for Total Knee Arthroplasty. Clin. Orthop. Relat. Res..

[10] Ethgen O., Bruyère O., Richy F., Dardennes C., Reginster J.-Y. (2004). Health-Related Quality of Life in Total Hip and Total Knee Arthroplasty. A Qualitative and Systematic Review of the Literature. J. Bone Jt. Surg. Am..

[11] Gerwin N., Hops C., Lucke A. (2006). Intraarticular Drug Delivery in Osteoarthritis. Adv. Drug Deliv. Rev..

[12] Creamer P. (1997). Intra-Articular Corticosteroid Injections in Osteoarthritis: Do They Work and If so, How?. Ann. Rheum. Dis..

[13] Piper S.L., Kramer J.D., Kim H.T., Feeley B.T. (2011). Effects of Local Anesthetics on Articular Cartilage. Am. J. Sports Med..

[14] Hartmann K., Koenen M., Schauer S., Wittig-Blaich S., Ahmad M., Baschant U., Tuckermann J.P. (2016). Molecular Actions of Glucocorticoids in Cartilage and Bone during Health, Disease, and Steroid Therapy. Physiol. Rev..

[15] Altman R.D., Manjoo A., Fierlinger A., Niazi F., Nicholls M. (2015). The Mechanism of Action for Hyaluronic Acid Treatment in the Osteoarthritic Knee: A Systematic Review. BMC Musculoskelet. Disord..

[16] Akmal M., Singh A., Anand A., Kesani A., Aslam N., Goodship A., Bentley G. (2005). The Effects of Hyaluronic Acid on Articular Chondrocytes. J. Bone Jt. Surg. Br..

[17] Greenberg D.D., Stoker A., Kane S., Cockrell M., Cook J.L. (2006). Biochemical Effects of Two Different Hyaluronic Acid Products in a Co-Culture Model of Osteoarthritis. Osteoarthr. Cartil..

[18] Hangody L., Szody R., Lukasik P., Zgadzaj W., Lénárt E., Dokoupilova E., Bichovsk D., Berta A., Vasarhelyi G., Ficzere A. (2018). Intraarticular Injection of a Cross-Linked Sodium Hyaluronate Combined with Triamcinolone Hexacetonide (Cingal) to Provide Symptomatic Relief of Osteoarthritis of the Knee: A Randomized, Double-Blind, Placebo-Controlled Multicenter Clinical Trial. Cartilage.

[19] Ozturk C., Atamaz F., Hepguler S., Argin M., Arkun R. (2006). The Safety and Efficacy of Intraarticular Hyaluronan with/without Corticosteroid in Knee Osteoarthritis: 1-Year, Single-Blind, Randomized Study. Rheumatol. Int..

[20] De Campos G.C., Rezende M.U., Pailo A.F., Frucchi R., Camargo O.P. (2013). Adding Triamcinolone Improves Viscosupplementation: A Randomized Clinical Trial. Clin. Orthop. Relat. Res..

[21] Caborn D., Rush J., Lanzer W., Parenti D., Murray C. (2004). A Randomized, Single-Blind Comparison of the Efficacy and Tolerability of Hylan G-F 20 and Triamcinolone Hexacetonide in Patients with Osteoarthritis of the Knee. J. Rheumatol..

[22] Livak K.J., Schmittgen T.D. (2001). Analysis of Relative Gene Expression Data Using Real-Time Quantitative PCR and the 2-ΔΔCT Method. Methods.

[23] Barbosa I., Garcia S., Barbier-Chassefière V., Caruelle J.P., Martelly I., Papy-García D. (2003). Improved and Simple Micro Assay for Sulfated Glycosaminoglycans Quantification in Biological Extracts and Its Use in Skin and Muscle Tissue Studies. Glycobiology.

[24] Fields J.K., Günther S., Sundberg E.J. (2019). Structural Basis of IL-1 Family Cytokine Signaling. Front. Immunol..

[25] Nees T.A., Rosshirt N., Zhang J.A., Reiner T., Sorbi R., Tripel E., Walker T., Schiltenwolf M., Hagmann S., Moradi B. (2019). Synovial Cytokines Significantly Correlate with Osteoarthritis-Related Knee Pain and Disability: Inflammatory Mediators of Potential Clinical Relevance. J. Clin. Med..

[26] Jin W., Dong C. (2013). IL-17 Cytokines in Immunity and Inflammation. Emerg. Microbes Infect..

[27] Mimpen J.Y., Baldwin M.J., Cribbs A.P., Philpott M., Carr A.J., Dakin S.G., Snelling S.J.B. (2021). Interleukin-17A Causes Osteoarthritis-Like Transcriptional Changes in Human Osteoarthritis-Derived Chondrocytes and Synovial Fibroblasts In Vitro. Front. Immunol..

[28] Mimpen J.Y., Carr A.J., Dakin S.G., Snelling S.J. (2018). Inhibition of Interleukin-17-Induced Effects in Osteoarthritis—An in Vitro Study. Osteoarthr. Cartil..

[29] Kongdang P., Chokchaitaweesuk C., Tangyuenyong S., Ongchai S. (2019). Proinflammatory Effects of IL-1β Combined with IL-17A Promoted Cartilage Degradation and Suppressed Genes Associated with Cartilage Matrix Synthesis In Vitro. Molecules.

[30] Schaefer E.C., Stewart A.A., Durgam S.S., Byron C.R., Stewart M.C. (2009). Effects of Sodium Hyaluronate and Triamcinolone Acetonide on Glucosaminoglycan Metabolism in Equine Articular Chondrocytes Treated with Interleukin-1. Am. J. Vet. Res..

[31] Bauer C., Niculescu-Morzsa E., Jeyakumar V., Kern D., Späth S.S., Nehrer S. (2016). Chondroprotective Effect of High-Molecular-Weight Hyaluronic Acid on Osteoarthritic Chondrocytes in a Co-Cultivation Inflammation Model with M1 Macrophages. J. Inflamm..

[32] Siengdee P., Radeerom T., Kuanoon S., Euppayo T., Pradit W., Chomdej S., Ongchai S., Nganvongpanit K. (2015). Effects of Corticosteroids and Their Combinations with Hyaluronanon on the Biochemical Properties of Porcine Cartilage Explants. BMC Vet. Res..

[33] Ayhan E., Kesmezacar H., Akgun I. (2014). Intraarticular Injections (Corticosteroid, Hyaluronic Acid, Platelet Rich Plasma) for the Knee Osteoarthritis. World J. Orthop..

[34] Song Y.W., Zhang T., Wang W.B. (2012). Gluococorticoid Could Influence Extracellular Matrix Synthesis through Sox9 via P38 MAPK Pathway. Rheumatol. Int..

[35] Rhee D.K., Marcelino J., Baker M., Gong Y., Smits P., Lefebvre V., Jay G.D., Stewart M., Wang H., Warman M.L. (2005). The Secreted Glycoprotein Lubricin Protects Cartilage Surfaces and Inhibits Synovial Cell Overgrowth. J. Clin. Investig..

[36] Richardson D.W., Dodge G.R. (2003). Dose-Dependent Effects of Corticosteroids on the Expression of Matrix-Related Genes in Normal and Cytokine-Treated Articular Chondrocytes. Inflamm. Res. Off. J. Eur. Histamine Res. Soc..

[37] Suntiparpluacha M., Tammachote N., Tammachote R. (2016). Triamcinolone Acetonide Reduces Viability, Induces Oxidative Stress, and Alters Gene Expressions of Human Chondrocytes. Eur. Rev. Med. Pharmacol. Sci..

[38] Wu P.-T., Kuo L.-C., Su F.-C., Chen S.-Y., Hsu T.-I., Li C.-Y., Tsai K.-J., Jou I.-M. (2017). High-Molecular-Weight Hyaluronic Acid Attenuated Matrix Metalloproteinase-1 and -3 Expression via CD44 in Tendinopathy. Sci. Rep..

[39] Coleman J.W. (2001). Nitric Oxide in Immunity and Inflammation. Int. Immunopharmacol..

[40] Liu T., Zhang L., Joo D., Sun S.C. (2017). NF-ΚB Signaling in Inflammation. Signal Transduct. Target. Ther..

[41] Aktan F. (2004). INOS-Mediated Nitric Oxide Production and Its Regulation. Life Sci..

[42] Campos S.B., Ashworth S.L., Wean S., Hosford M., Sandoval R.M., Hallett M.A., Atkinson S.J., Molitoris B.A. (2009). Cytokine-Induced F-Actin Reorganization in Endothelial Cells Involves RhoA Activation. Am. J. Physiol. Ren. Physiol..

[43] Chen C., Xie J., Rajappa R., Deng L., Fredberg J., Yang L. (2015). Interleukin-1β and Tumor Necrosis Factor-α Increase Stiffness and Impair Contractile Function of Articular Chondrocytes. Acta Biochim. Biophys. Sin..

[44] Haudenschild D.R., Chen J., Steklov N., Lotz M.K., D’Lima D.D. (2009). Characterization of the Chondrocyte Actin Cytoskeleton in Living Three-Dimensional Culture: Response to Anabolic and Catabolic Stimuli. Mol. Cell. Biomech..

[45] Yourek G., Hussain M.A., Mao J.J. (2007). Cytoskeletal Changes of Mesenchymal Stem Cells during Differentiation. ASAIO J..

[46] Vorselen D., Roos W.H., MacKintosh F.C., Wuite G.J.L., van Loon J.J.W.A. (2014). The Role of the Cytoskeleton in Sensing Changes in Gravity by Nonspecialized Cells. FASEB J. Off. Publ. Fed. Am. Soc. Exp. Biol..

[47] Nemirovskiy O.V., Radabaugh M.R., Aggarwal P., Funckes-Shippy C.L., Mnich S.J., Meyer D.M., Sunyer T., Rodney Mathews W., Misko T.P. (2009). Plasma 3-Nitrotyrosine Is a Biomarker in Animal Models of Arthritis: Pharmacological Dissection of INOS’ Role in Disease. Nitric Oxide Biol. Chem..

[48] Lepetsos P., Papavassiliou A.G. (2016). ROS/Oxidative Stress Signaling in Osteoarthritis. Biochim. Biophys. Acta.

[49] Zahan O.-M., Serban O., Gherman C., Fodor D. (2020). The Evaluation of Oxidative Stress in Osteoarthritis. Med. Pharm. Rep..

[50] Li D., Xie G., Wang W. (2012). Reactive Oxygen Species: The 2-Edged Sword of Osteoarthritis. Am. J. Med. Sci..

[51] Henrotin Y., Kurz B., Aigner T. (2005). Oxygen and Reactive Oxygen Species in Cartilage Degradation: Friends or Foes?. Osteoarthr. Cartil..

[52] Wernecke C., Braun H.J., Dragoo J.L. (2015). The Effect of Intra-Articular Corticosteroids on Articular Cartilage: A Systematic Review. Orthop. J. Sport. Med..

[53] Wang F., He X. (2015). Intra-Articular Hyaluronic Acid and Corticosteroids in the Treatment of Knee Osteoarthritis: A Meta-Analysis. Exp. Ther. Med..

[54] Wang C.-P., Lee W.-C., Hsieh R.-L. (2021). Effects of Repeated Co-Injections of Corticosteroids and Hyaluronic Acid on Knee Osteoarthritis: A Prospective, Double-Blind Randomized Controlled Trial. Am. J. Med..

[55] Smith C., Patel R., Vannabouathong C., Sales B., Rabinovich A., McCormack R., Belzile E.L., Bhandari M. (2019). Combined Intra-Articular Injection of Corticosteroid and Hyaluronic Acid Reduces Pain Compared to Hyaluronic Acid Alone in the Treatment of Knee Osteoarthritis. Knee Surg. Sports Traumatol. Arthrosc..

[56] Von der Mark K., Gauss V., von der Mark H., Müller P. (1977). Relationship between Cell Shape and Type of Collagen Synthesised as Chondrocytes Lose Their Cartilage Phenotype in Culture. Nature.

